# Supplementary material for: Subglacial water amplifies Antarctic contributions to sea-level rise
Source: Nat Commun. 2025 Apr 7;16:3187. doi: 10.1038/s41467-025-58375-4 (PMC11976955; doi:10.1038/s41467-025-58375-4)
Supplement: Supplementary file 1 — Supplementary Information [file 41467_2025_58375_MOESM1_ESM.pdf]

Supplementary Information for

Subglacial Water Amplifies Antarctic Contributions to Sea-Level Rise

Chen Zhao[1*,2], Rupert Gladstone[3], Thomas Zwinger[4], Fabien Gillet-Chaulet[5], Yu Wang[1], Justine Caillet[5], Pierre Mathiot[5], Leopekka Saraste[4], Ben Galton-Fenzi[5,1,2], Poul Christoffersen[7,1,2], and Matt A. King[2]

[1*]Australian Antarctic Program Partnership,Institute for Marine and Antarctic Studies, University of Tasmania, Hobart, Australia
[2]Australian Centre for Excellence in Antarctic Science, University of Tasmania, Hobart, Australia
[3]Arctic Centre, University of Lapland, Rovaniemi, Finland
[4]CSC-IT Center for Science, Espoo, Finland
[5]Institut des Géosciences et de l'Environnement, University Grenoble, Grenoble, France
[6]Australian Antarctic Division, Hobart, Australia
[7]Institute for Marine and Antarctic Studies, University of Tasmania, Hobart, Australia
[*]chen.zhao@utas.edu.au

Inversion analysis in model configuration

We follow ref.[1] to perform an L surface analysis to determine the optimal values for λ_β, $\lambda_{E_\eta 1}$ and $\lambda_{E_\eta 2}$. Utilizing an empirical value, we set $\lambda_{E_\eta 2}$ to 0.02. We initialise the remaining two regularisation parameters λ_β and $\lambda_{E_\eta 1}$ with an initial guess, i.e. an exponential sequence with a common ratio of 10. As shown in Fig. S1, we were able to narrow down the feasible range for λ_β and $\lambda_{E_\eta 1}$ to conduct a following L surface tests. As shown in Fig S2, $\lambda_\beta = 1e5$ and $\lambda_{E_\eta 1} = 1e6$ is the point provides the best fit based on visual perception. The simulated basal drag and viscosity with the optimal λ_β and $\lambda_{E_\eta 1}$ is shown in Fig. S3;

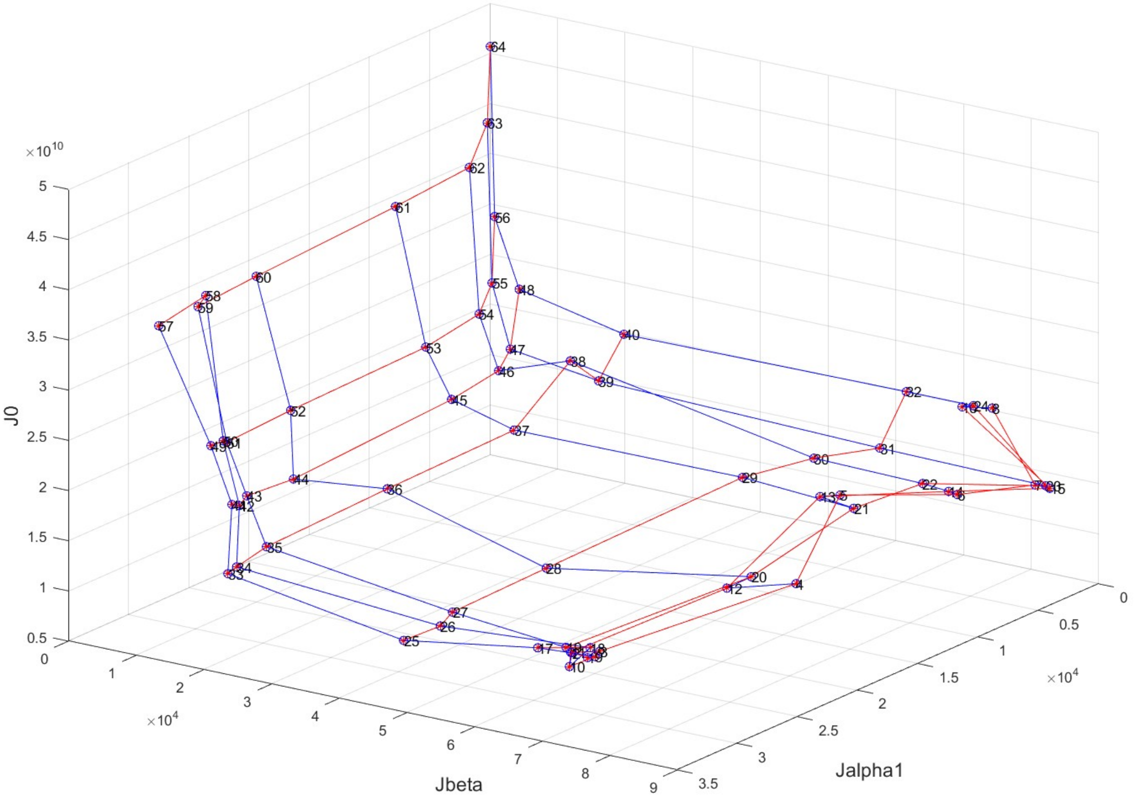

Figure S1. L surface analysis. The red line connects points with the same λ_β, while the blue line connects points with the same $\lambda_{E_\eta 1}$.

Sensitivity analysis to various climate models

Under the high emission scenario, the UKESM model from the Coupled Model Intercomparison Project phase 6 (CMIP6[2]) projects the highest sea level contribution of nearly 2 m by 2300 with a RC relation, while the same model with LW shows a negative contribution of 166 mm. CESM2, another CMIP6 climate model, forecasts the second highest sea-level rise contribution of 1614 mm, which is nearly 10 times greater than its output using with LW relation. The HadGEM2 model from CMIP5, using a RC relation, estimates a sea level contribution of 1260 mm, whereas with the LW relation, it indicates a contribution of 288 mm. The remaining two climate models, NorESM1 and CCSM4, offer the lowest contributions under a RC relation (470 mm and 188 mm, respectively). However, both models show negative sea level contributions with the LW relation. These significant differences across climate models are primarily driven largely driven by variations in surface mass balance and basal mass balance constrained by these climate models (Fig. S10).

Under the low emission scenario, the UKEMS model with a LW sliding relation suggests a negative sea-level rise contribution of 255 mm while the NorESM1-M model indicates a positive contribution of 137 mm. With a RC relatioin, both climate models predicts a near-balance by 2300, with NorESM-1 and UKESM indicating a sea-level rise contribution of 16 mm

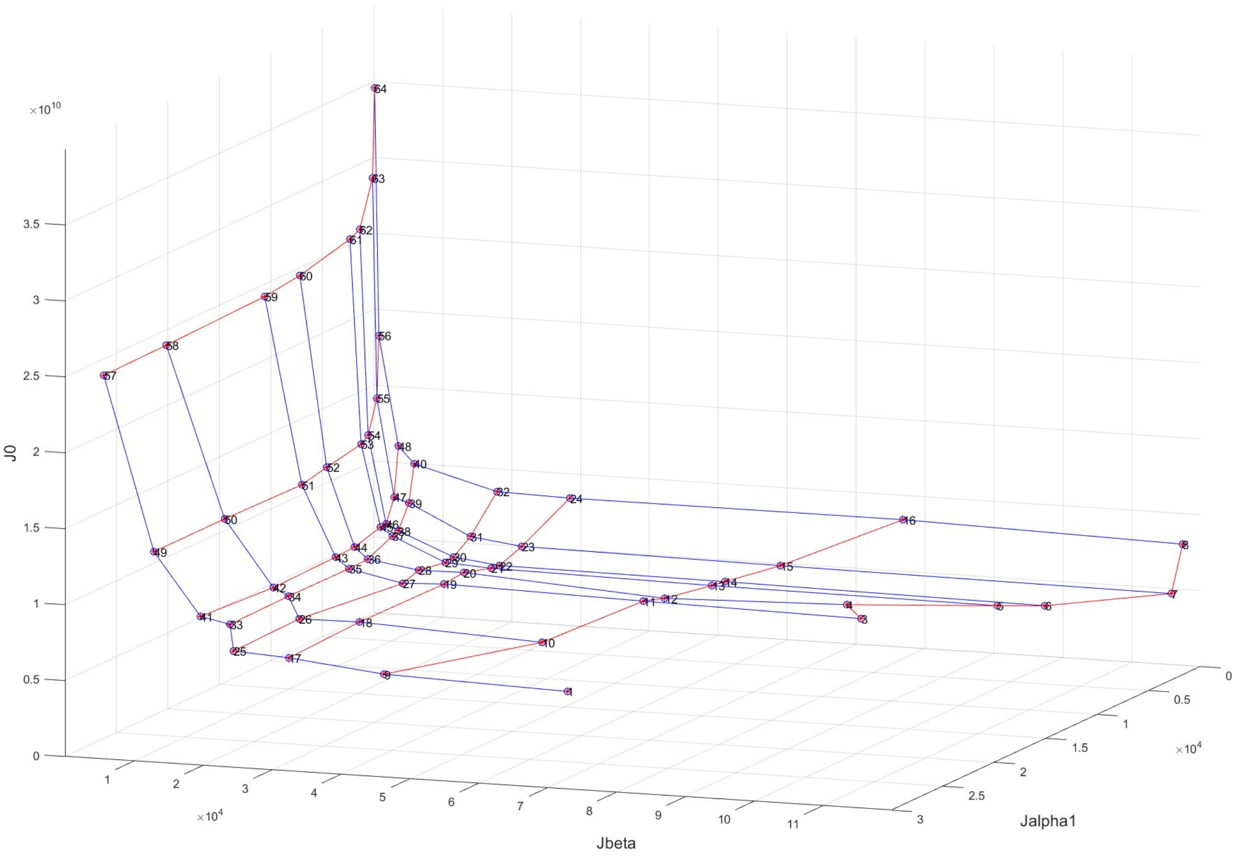

Figure S2. L surface analysis. The red line connects points with the same λ_β, while the blue line connects points with the same $\lambda_{E_\eta 1}$.

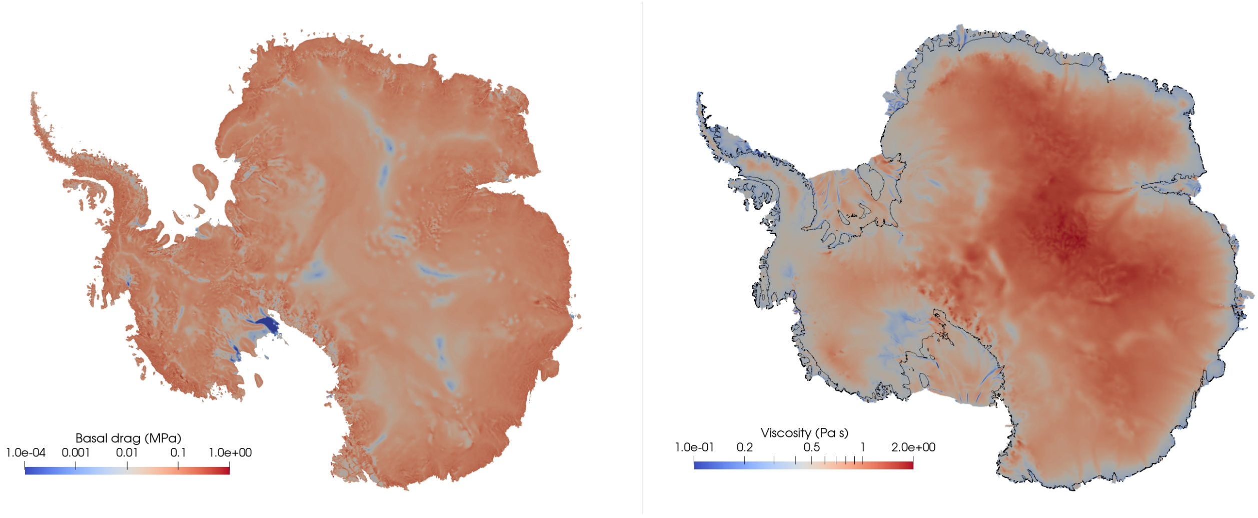

Figure S3. Simulated (a) basal drag and (b) ice viscosity from the inversion.

and -7 mm, respectively. The UKESM-High model with RC relation predicts a sea-level contribution of 2 m by 2300 while LW shows mass gain. Among five climate models, CCSM4-High reveals the lowest ice mass loss under both low and high emission scenarios, correlating with higher surface accumulation rates further inland (Fig. S10).

Basin-scale analysis

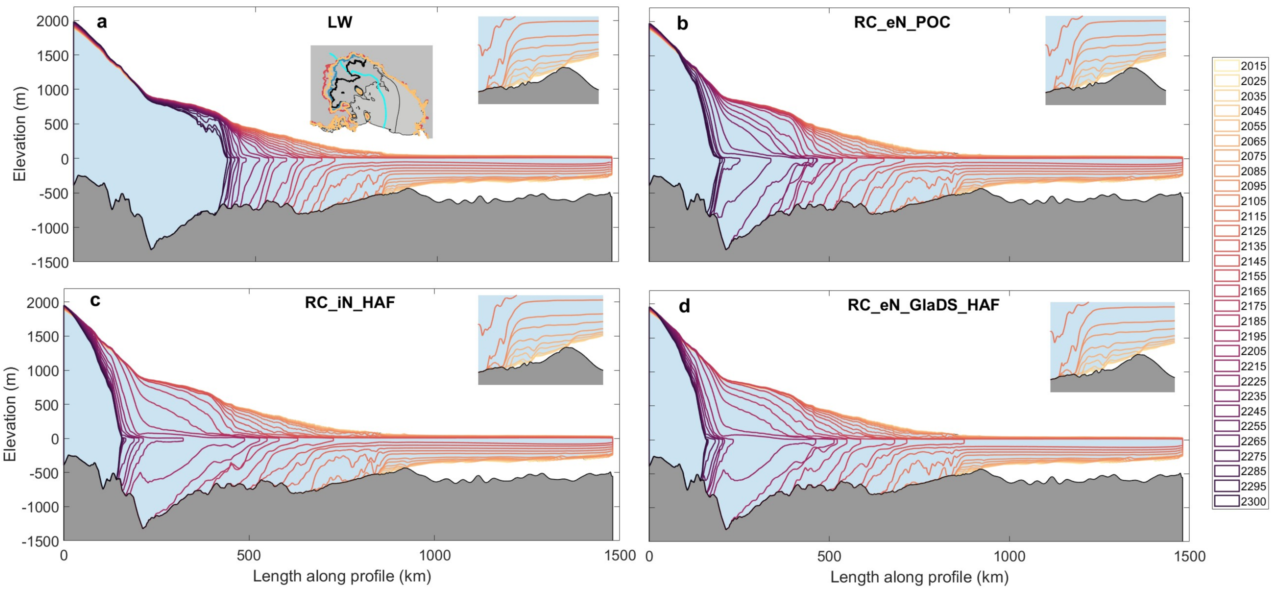

Figure S4. Time series (2015-2300) of grounding line position along the flowline (cyon color in the inset) of Whillans Stream from the Siple Coast in Basin E-Ep. The colored dots in the inset of panel (a) show the grounding line position in 2300 from **LW** (light grey), **RC_iN_HAF** (purple), **RC_eN_POC** (blue), and **RC_eN_GlaDS_HAF** (red).

Basin Ep-F in West Antarctica, which feeds the Ross Ice Shelf with six ice streams, exhibits substantial GL flux increases and dramatic GL retreat after 2100 under all experiments (Fig. S4). The extreme scenario with implicit N and HAF-scaling first attains its maximum increase (2130 $\mathrm{Gt\,yr^{-1}}$) in 2194, followed closely by **RC_eN_GlaDS_HAF** reaching 1815 $\mathrm{Gt\,yr^{-1}}$ in 2197. Notably, **RC_iN_HAF** shows a sharp GL flux surge around 2250 and 2280, linked to rapid retreat of ice streams D and E (Fig. S4a inset). By 2300, experiments with HAF-scaling show similar GL flux increases and positions, while **RC_eN_POC** exhibits the least mass loss with a GL position approximately 20 km behind.

In the Wilkes Subglacial Basin (Basin D-Dp), equivalent to 3 to 4 meters of SLR, **RC_iN_HAF** experiences accelerated GL flux after 2120, stabilizing until 2267 before a pronounced surge, indicating tipping points with rapid GL retreat (Fig. S5c). **RC_eN_POC** initiates an acceleration in ice mass loss from 2200, intensifying further around 2250, and surging again near 2290. By 2300, the GL flux increase from **RC_iN_HAF** nearly doubles that of **RC_eN_POC**, four times that of **RC_eN_GlaDS_HAF**, and exceeds **RC_eN_GlaDS** by by over fivefold, reflecting differences in GL positions by 2300 (Fig. S5).

The Aurora Subglacial Basin (Basin Cp-D), feeding the Totten catchment in East Antarcitca with 3.5 m of SLR equivalent, displays similar GL flux patterns across all RC experiments, peaking between 2170 and 2190 and gradually decreasing thereafter until 2300. The experiment with constant N from GlaDS shows a delayed peak around 2215. By 2300, GL flux ranges between 250-326 $\mathrm{Gt\,yr^{-1}}$, with consistent GL movements and minor timing differences (Fig. S5).

Sensitivity to threshold h_T used in HAF-scaling

Ice dynamics is highly sensitive to the threshold h_T used in HAF-scaling, which determines the extent of reduction in basal drag above the GL[3]. This scaling assumes a transition from unmodified drag upstream to no drag at the GL where the ice is afloat. A threshold value of 75 m for the PIG was adopted based on earlier analysis[4] and supported by simulated data for the whole Antarctic in this study (see Methods). However, subsequent research on PIG suggests a value of 46 m may better align with 15 years of observations[5], though its applicability may vary across Antarctic regions and time scales. The initial estimation of h_T can be guided by the basal drag-HAF relationship but must be carefully assessed for individual glaciers and updated over time due to potential significant changes in ice geometry. Separate tests using h_T = 46 m in **RC_iN_HAF46** confirm the considerable sensitivity to the chosen threshold in HAF-scaling, while **RC_eN_GlaDS_HAF46** shows less sensitivity (Fig. S17, Fig. S16). Notably, different basins exhibit varying sensitivity to the threshold (Fig. S16). For some glaciers, like Thwaites Glacier and Cook Glacier, the 46 m and 75 m HAF contours are closely positioned (Fig. S13). This indicates that the weakened area can be highly influenced by GL mesh resolution, with only one or two mesh elements separating them (S13).

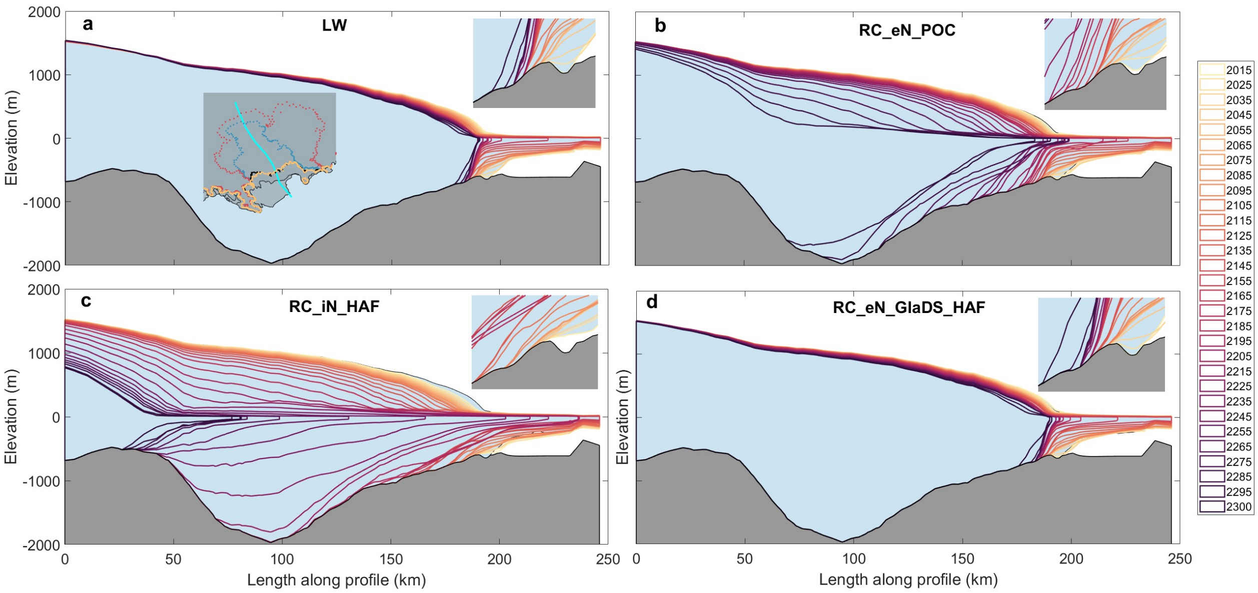

Figure S5. Time series (2015-2300) of grounding line position along the flowline (cyon color in the inset) of Cook Glacier from Wilkes Subglacial Basin. The colored dots in the inset of panel (a) show the grounding line position in 2300 from **LW** (light grey), **RC_iN_HAF** (purple), **RC_eN_POC** (blue), and **RC_eN_GlaDS_HAF** (red).

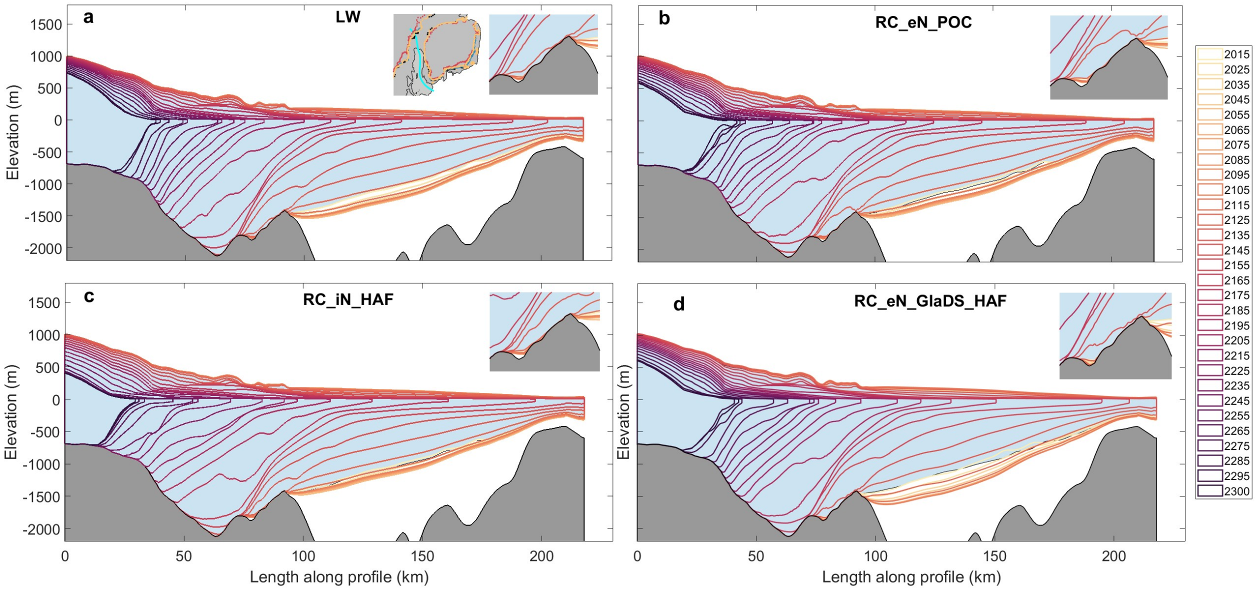

Figure S6. Time series (2015-2300) of grounding line position along the flowline (cyon color in the inset) of Totten Glacier from Aurora Subglacial Basin. The colored dots in the inset of panel (a) show the grounding line position in 2300 from **LW** (light grey), **RC_iN_HAF** (purple), **RC_eN_POC** (blue), and **RC_eN_GlaDS_HAF** (red).

Extended Figures and Tables

ISMIP6-2100[6]		ISMIP6-2300[7]		ABUMIP[8]	
**Model Name**	**Sliding Law**	**Model Name**	**Sliding Law**	**Model Name**	**Sliding Law**
AWI_PISM	Mohr-Coulomb (m=3)	-	-	AWI_PISMPal	Coulomb
PIK_PISM	Mohr-Coulomb (m=3)	PIK_PISM	Mohr-Coulomb (m=3)	PIK_PISM	Coulomb
VUW_PISM	Pseudo-plastic	VUW_PISM	Pseudo-plastic	-	-
-	-	-	-	ARC_PISM	Coulomb
-	-	UCM_Yelmo	Regularised Coulomb	-	-
IMAU_IMAUICE	Weertman(m=3)	IMAU_UFEMISM	Regularised Coulomb	IMAU_ICE	Coulomb
NCAR_CISM	Weertman(m=3)–Coulomb	NCAR_CISM	Zoet-Iversen	NCAR_CISM	Weertman(m=3)–Coulomb
-	-	NORCE_CISM	Zoet-Iversen	-	-
DOE_MALI	Weertman(m=1)	DOE_MALI	Weertman (m=3)	DOE_MALI	Weertman(m=1)
ILTS_PIK_SICOPOLIS	Weertman(m=3)–Budd	ILTS_PIK_SICOPOLIS	Weertman(m=3)–Budd	ILTS_PIK_SICOPOLIS	Weertman(m=3)
JPL_ISSM	Budd(m=1)	-	-	JPL_ISSM	Weertman(m=1)
-	-	UCSD_ISSM	Weertman(m=3)–Budd	-	-
UCIJPL_ISSM	Weertman(m=3)	DC_ISSM	Budd	-	-
ULB_FETISH	Weertman(m=2)	ULB_Kori	Weertman(m=3)	ULB_FETISH	Weertman(m=2)
UTAS_ELMERICE	Weertman(m=1)	UTAS_ELMERICE	Weertman (m=1)	-	-
-	-	IGE_ElmerIce	Weertman (m=1)	IGE_ElmerIce	Weertman (m=3)
-	-	UNN_Úa	Weertman(m=3)	-	-
VUB_AISMPALEO	Weertman(m=3)	VUB_AISMPALEO	Weertman (m=3)	-	-
LCSE_GRISLI	Weertman(m=3)	LCSE_GRISLI	Weertman(m=3)	LCSE_GRISLI	Coulomb
-	-	-	-	CPOM_BISICLES	Weertman(m=3)–Coulomb
-	-	-	-	PSU_PSU3D	Weertman(m=2)

Table S1. Sliding law used in ISMIP6-2100 Antarctic[6], ISMIP6-2300 Antarctic[7], and ABUMIP[8].

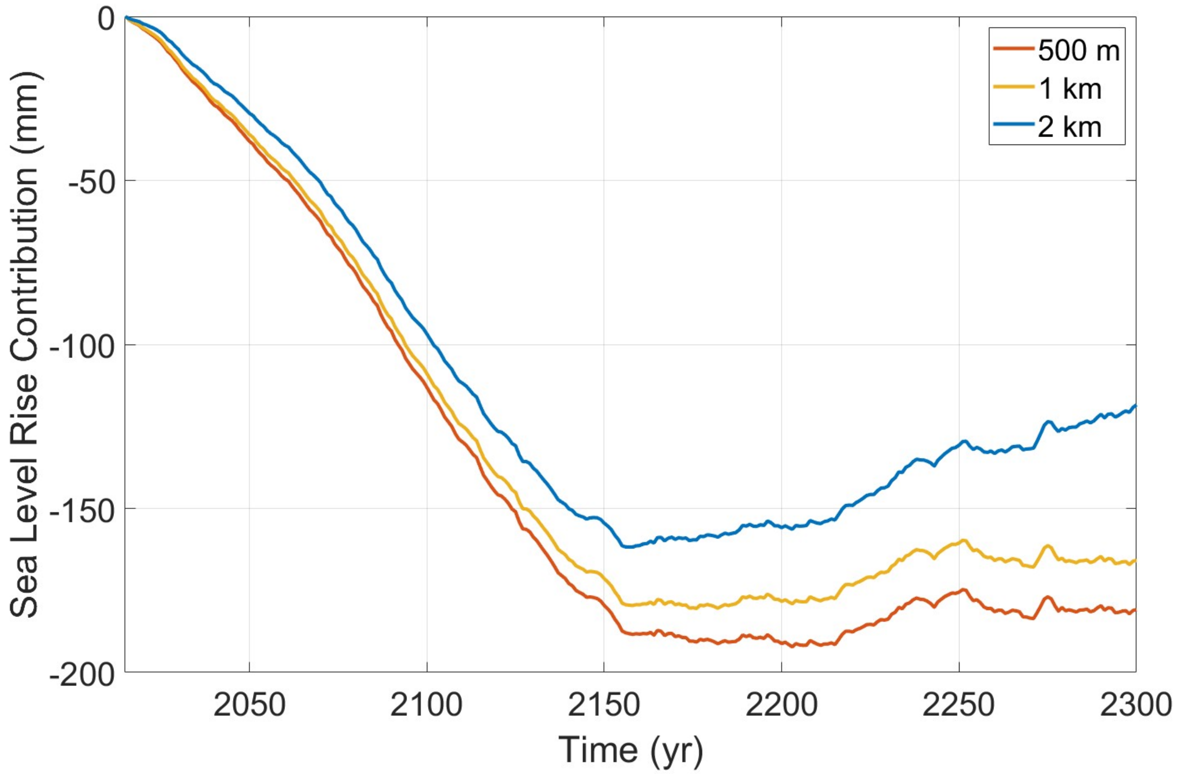

Figure S7. Sensitivity of sea-level contributions with linear Weertman law to various mesh resolutions. Higher-resolution meshes predict less mass loss compared to coarser resolutions.

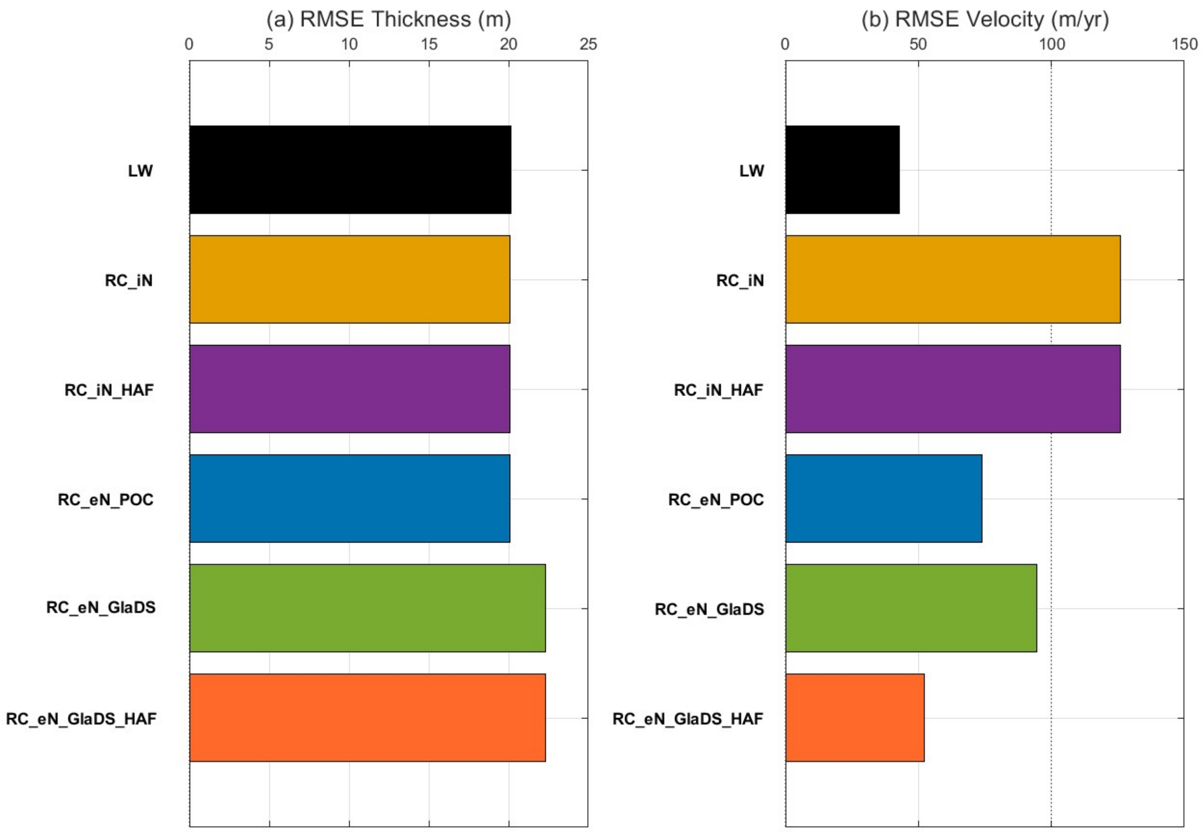

Figure S8. Root Mean Square Error (RMSE) between the observed and modeled ice thickness (a, in m) and surface ice velocity in 1995 (b, in $m\,yr^{-1}$). Observations from BedMachine v3 [9] and MEaSUREs Phase-Based Antarctica Ice Velocity map (Version 1) [10] datasets were interpolated to the mesh nodes for calculation.

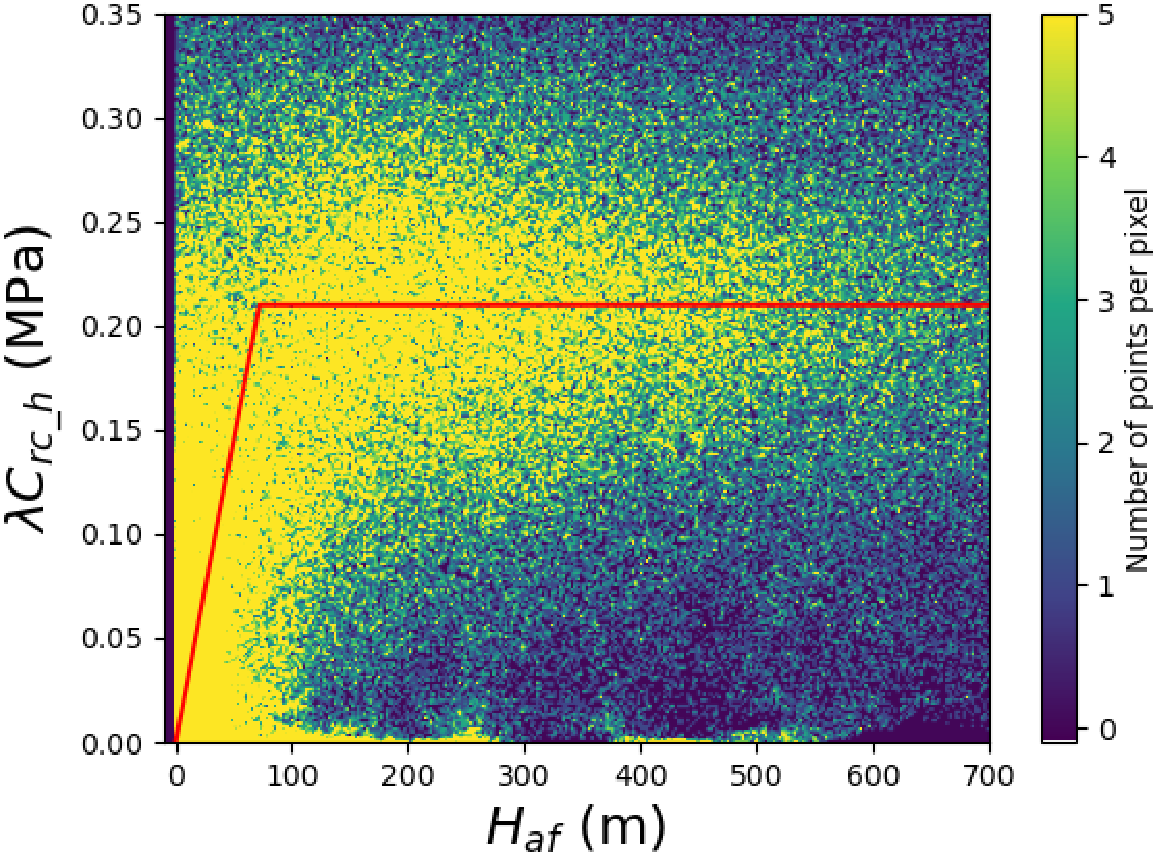

Figure S9. Correlation between the converted $\lambda \cdot C_2$ and the height above flotation, h_{af}, based on the initial geometry. The red line represents a visually linear fit that suggests $\lambda \cdot C_2$ tends to decrease linearly where $h_{af} < 75m$ while $\lambda \cdot C_2$ keeps constant where $h_{af} > 75m$.

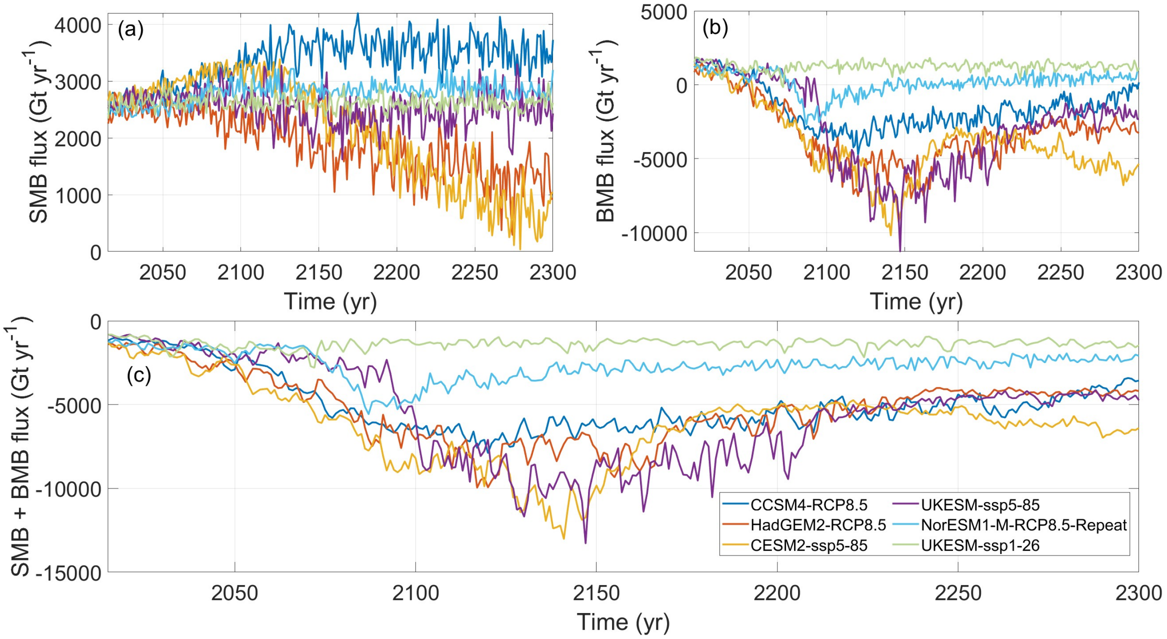

Figure S10. (a) Surface mass balance (SMB) (b) basal mass balance (BMB), and (c) the sum of SMB and BMB over 2015 to 2300 from experiments with different climate models.

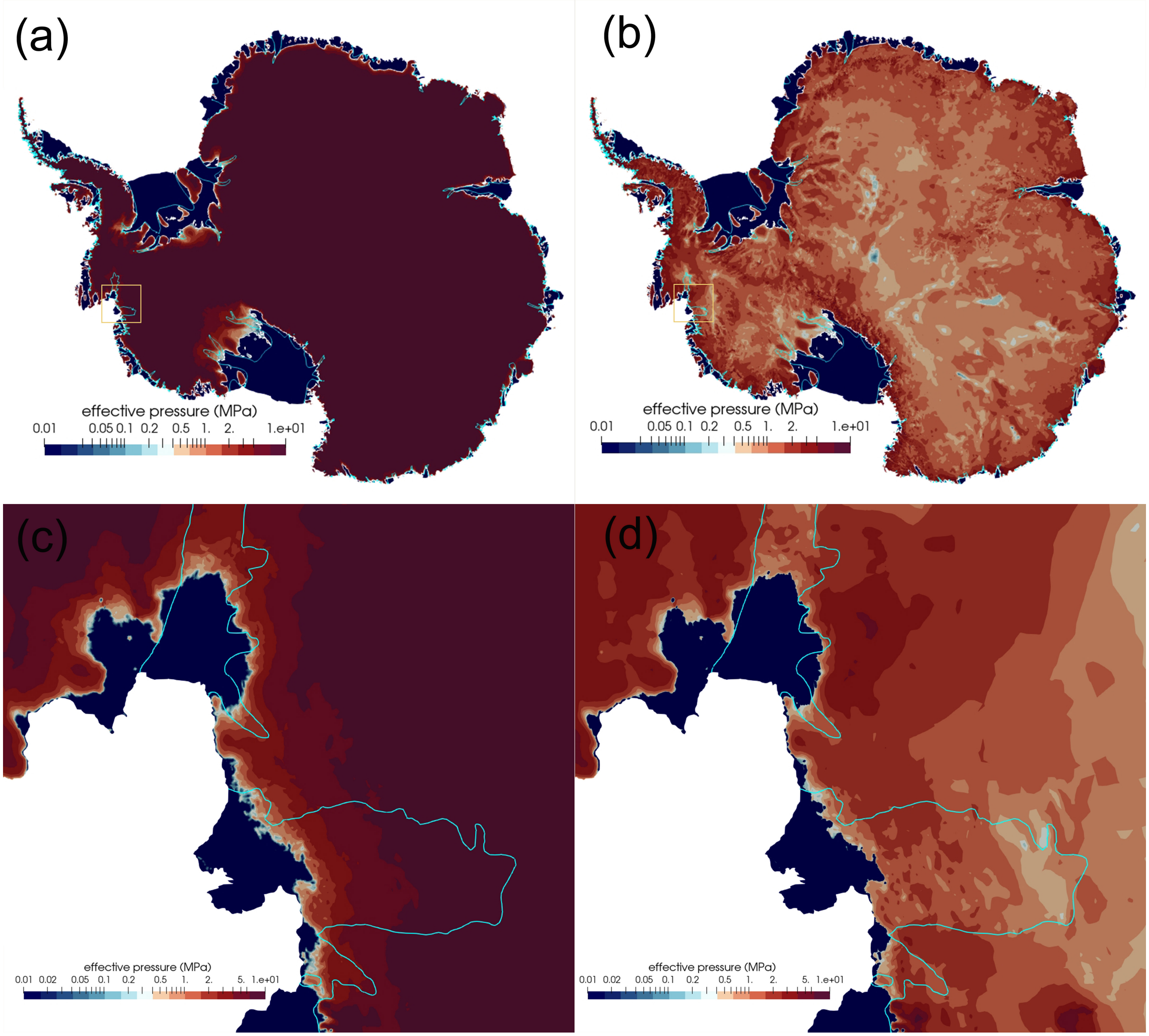

Figure S11. Effective pressure at year 1995 from (a,c) **RC_eN_POC** and (b,d) **RC_eN_GlaDS_HAF**. The blue line is the velocity contour of 300 $\mathrm{m\,yr^{-1}}$.

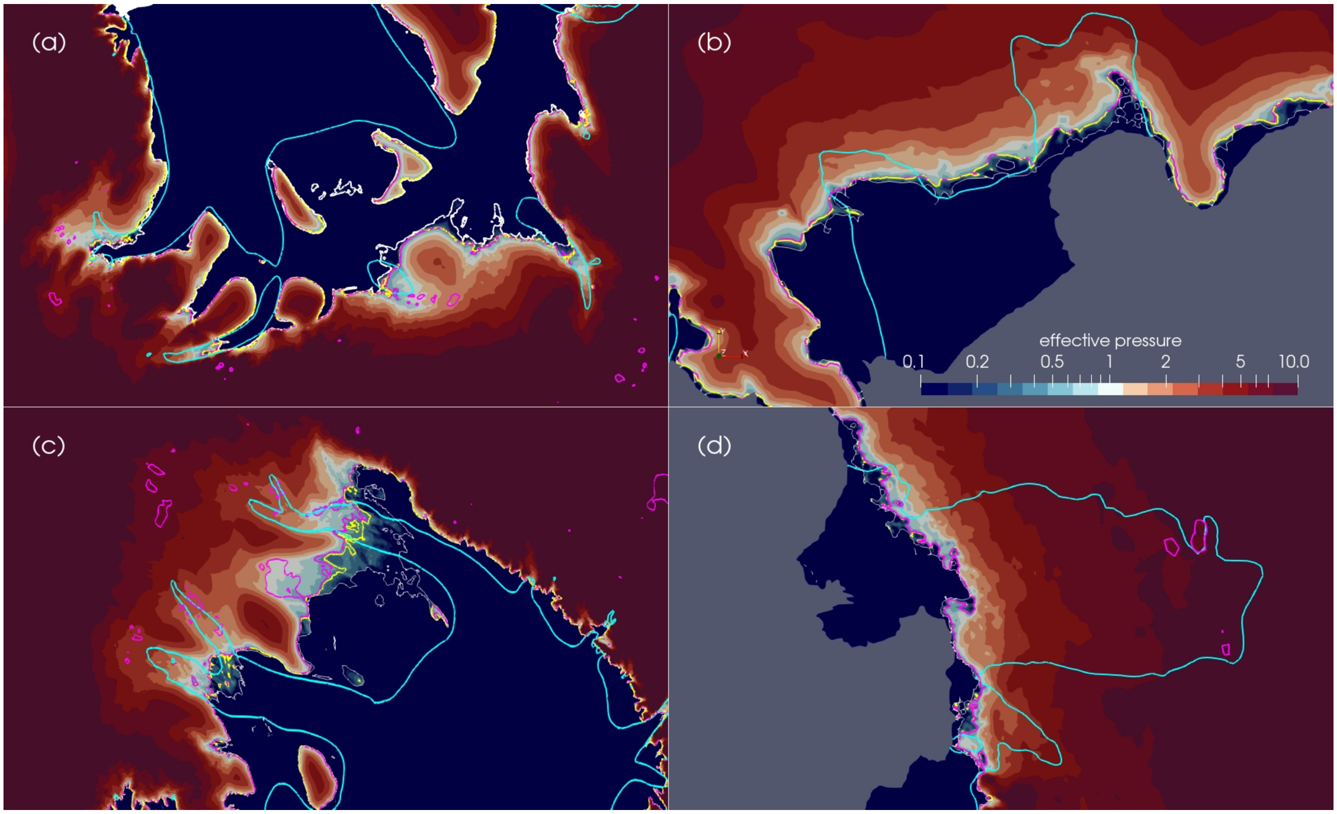

Figure S12. Coulomb limit from **RC_eN_POC** (yellow line), **RC_eN_GlaDS** (pink line) with the background from the effective pressure at year 1995 from **RC_eN_POC**. The blue line is the velocity contour of 300 $\mathrm{m\,yr^{-1}}$ and the white line is the grounding line at 1995. The subdomain is (a) glacier feeding the Filchner-Ronne Ice Shelf, (b) Cook Glacier, (c) Siple Coast and (d) Thwaites Glacier.

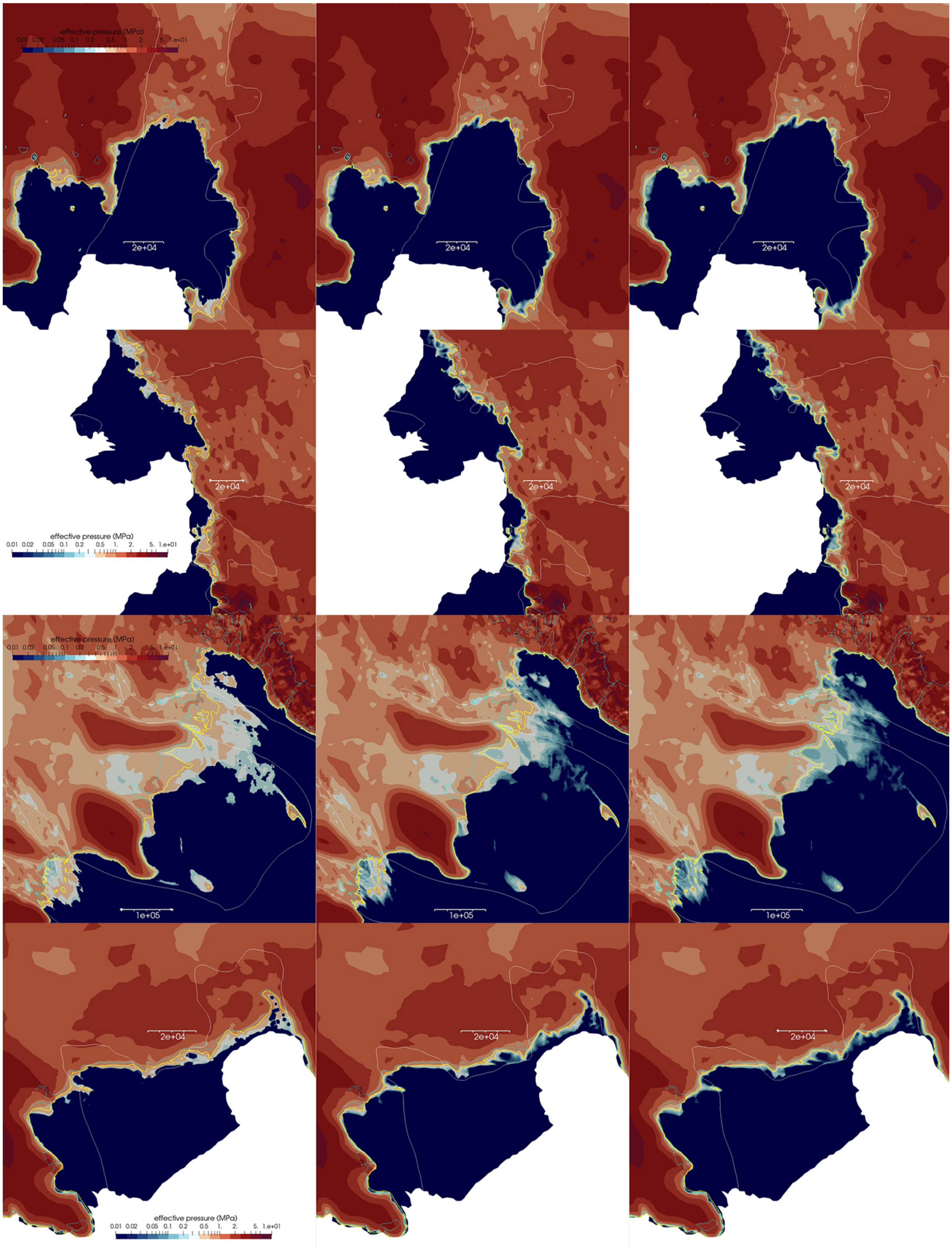

Figure S13. Effective pressure at year 1995 from **RC_eN_GlaDS** (left column), **RC_eN_GlaDS_HAF** ht = 46 m (middle column), and **RC_eN_GlaDS_HAF** ht = 75 m (right column) for Pine Island Glacier (first row), Thwaites Glacier (second row), Sidle Coast (third row), and Cook Glacier (last row). The white line is the velocity contour of 300 $\mathrm{m\,yr^{-1}}$. The yellow and blue lines are the 46 m and 75 m contours of height above flotation in 1995.

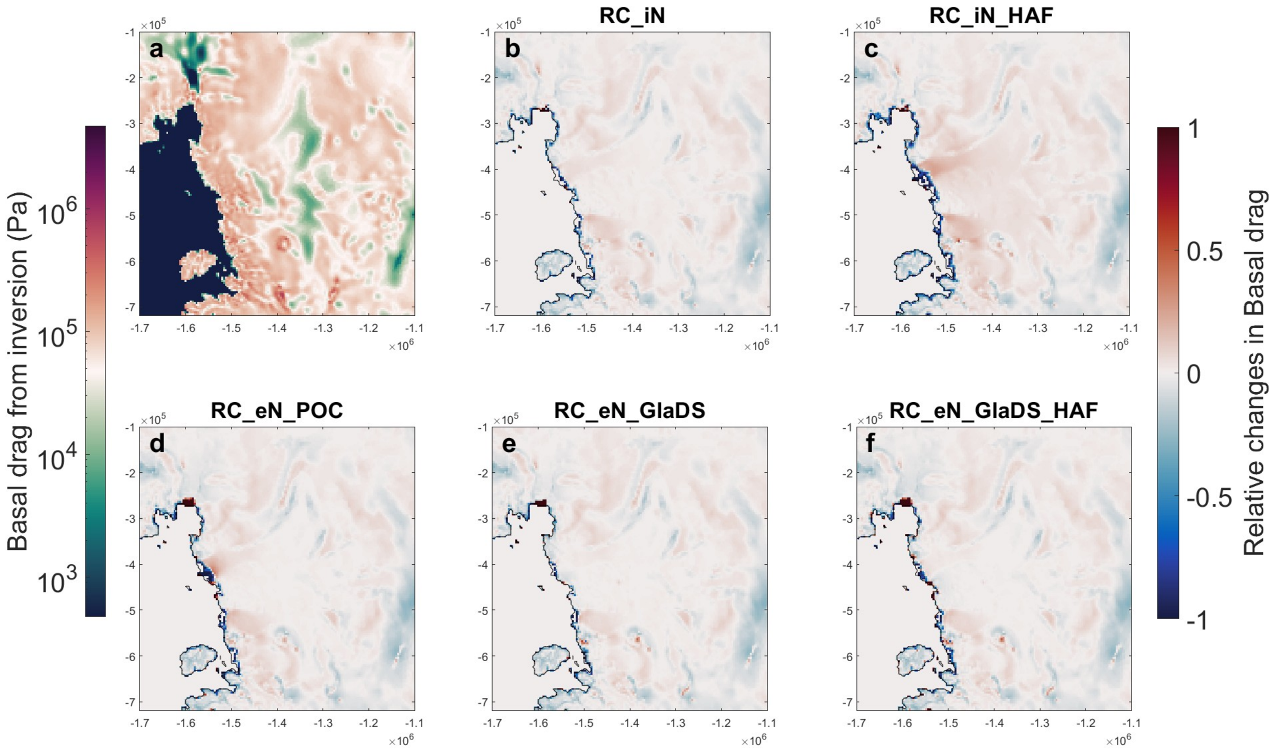

Figure S14. (a) Inverted basal drag (Pa). Relative changes in basal drag from (b) **RC_iN**, (c) **RC_iN_HAF**, (d) **RC_eN_POC**, (e) **RC_eN_GlaDS**, and (f) **RC_eN_GlaDS_HAF** in year 2015 compared with the inverted basal drag. Positive means more basal drag than the inverted. The black line shows the grounding line position in 1995.

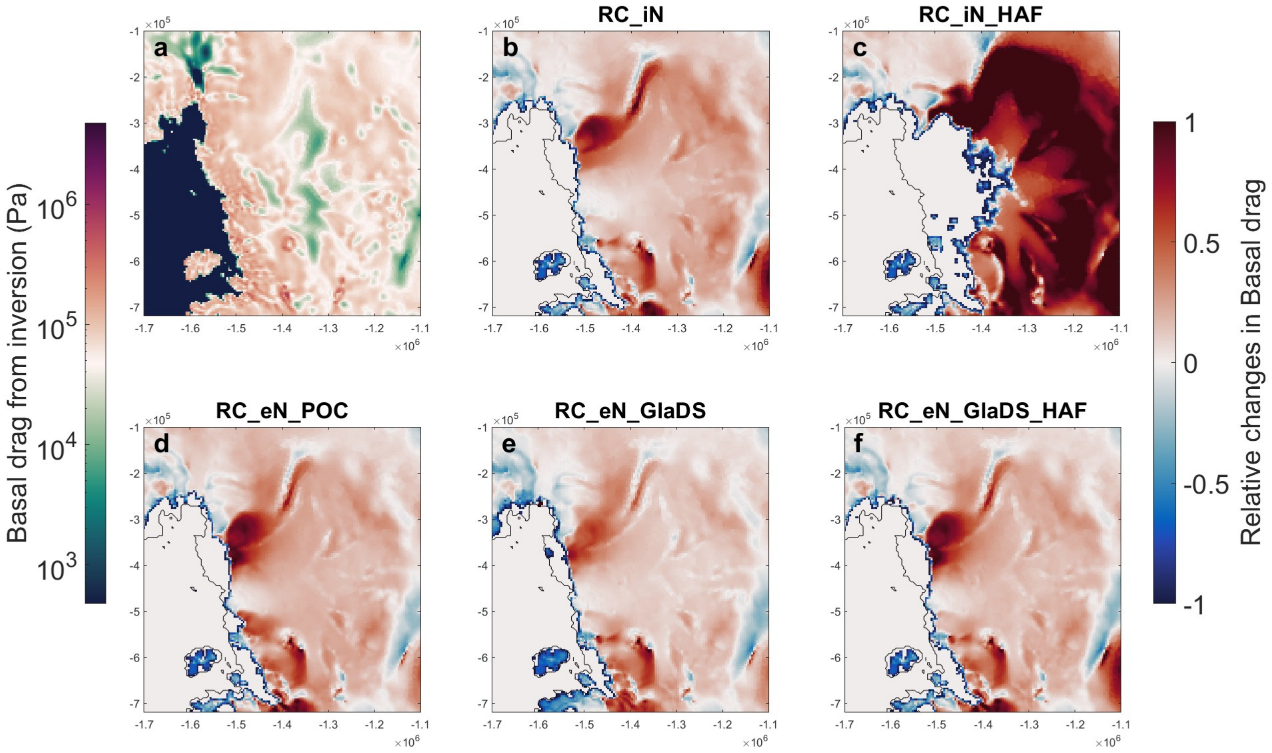

Figure S15. (a) Inverted basal drag (Pa). Relative changes in basal drag from (b) **RC_iN**, (c) **RC_iN_HAF**, (d) **RC_eN_POC**, (e) **RC_eN_GlaDS**, and (f) **RC_eN_GlaDS_HAF** in year 2270 compared with the inverted basal drag. Positive means more basal drag than the inverted. The black line shows the grounding line position in 1995.

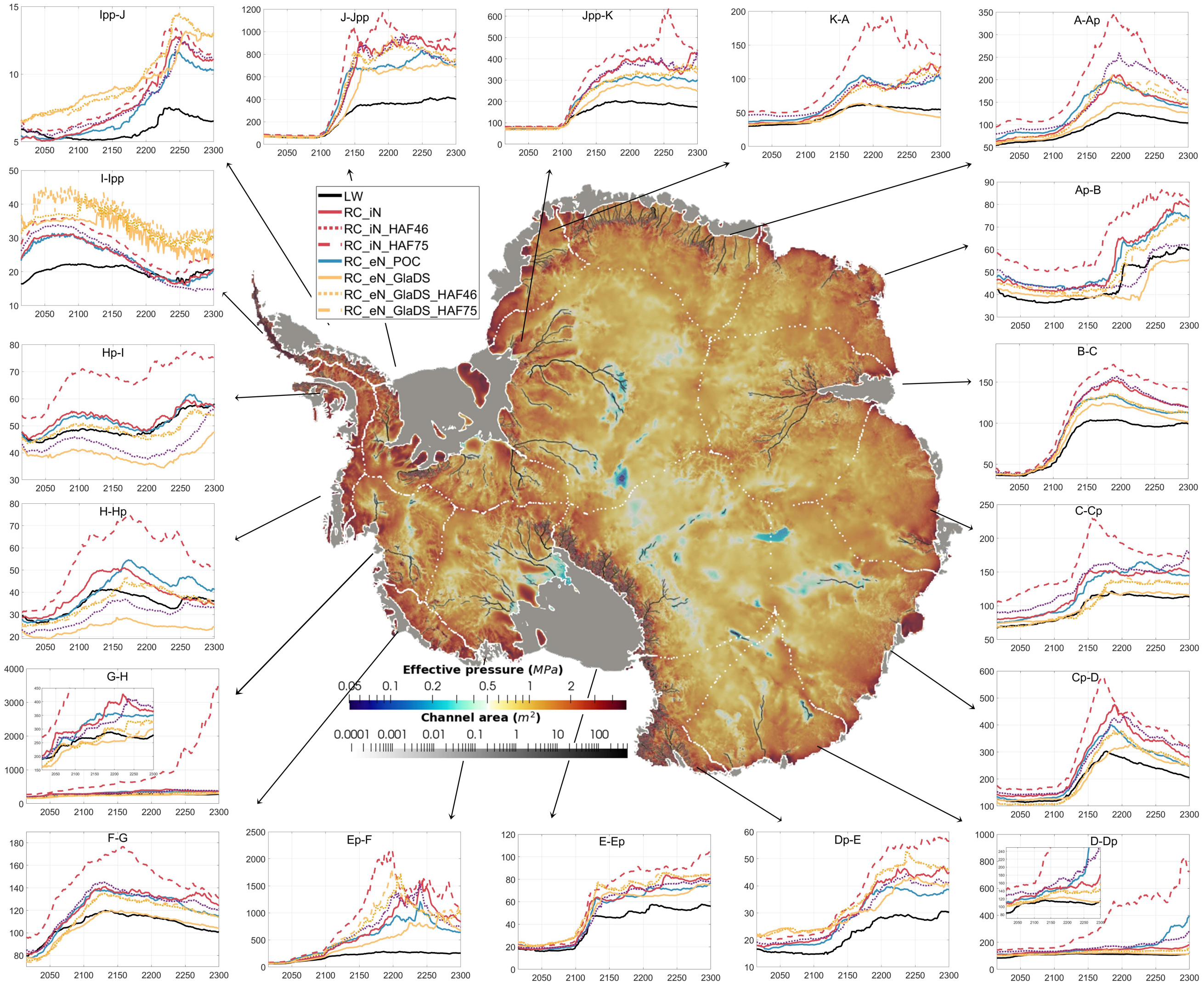

Figure S16. Time series (2015-2300) of grounding line flux (Gt yr^{-1}) across various basins under different experiments. The background image is the simulated effective pressure and channel area (cross-sectional area of each channel) from GlaDS. Y axis is the grounding line flux (unit: Gt yr^{-1}) and X axis is the time (unit: year).The Antarctic drainage basins are from E. Rignot and J. Mouginot[11]

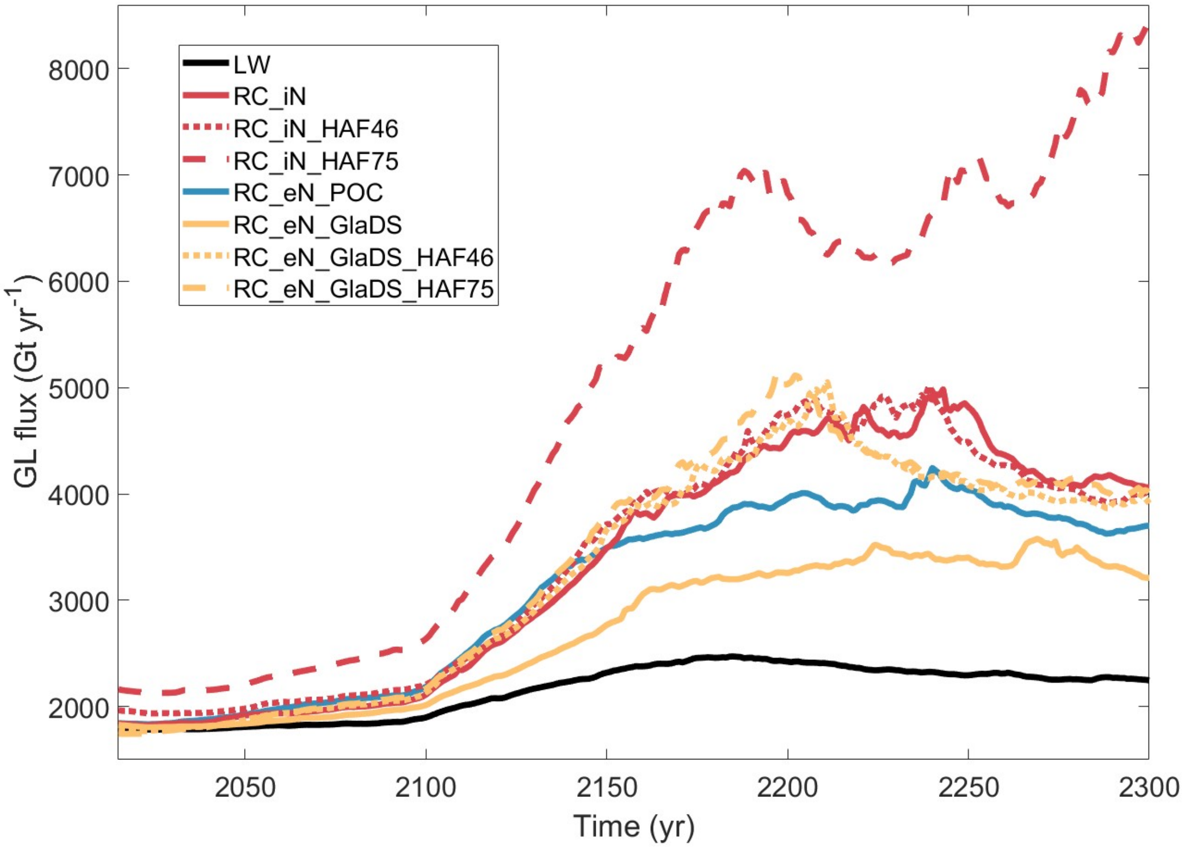

Figure S17. Time series (2015-2300) of grounding line flux for the Antarctic Ice Sheet under different experiments.

Supplementary References

1. Wang, Y. *et al.* Sensitivity of Future Projections of the Wilkes Subglacial Basin Ice Sheet to Grounding Line Melt Parameterizations. *EGUsphere* **2024**, 1–31, DOI: 10.5194/egusphere-2024-1005 (2024).
2. Nowicki, S. M. J. *et al.* Ice Sheet Model Intercomparison Project (ISMIP6) contribution to CMIP6. *Geosci. Model. Dev.* **9**, 4521–4545, DOI: 10.5194/gmd-9-4521-2016 (2016).
3. Joughin, I., Shapero, D. & Dutrieux, P. Responses of the Pine Island and Thwaites glaciers to melt and sliding parameterizations. *The Cryosphere* **18**, 2583–2601, DOI: 10.5194/tc-18-2583-2024 (2024).
4. Joughin, I., Smith, B. E. & Holland, D. M. Sensitivity of 21st century sea level to ocean-induced thinning of Pine Island Glacier, Antarctica. *Geophys. Res. Lett.* **37**, DOI: https://doi.org/10.1029/2010GL044819 (2010).
5. Joughin, I., Smith, B. E. & Schoof, C. G. Regularized Coulomb Friction Laws for Ice Sheet Sliding: Application to Pine Island Glacier, Antarctica. *Geophys. Res. Lett.* **46**, 4764–4771, DOI: https://doi.org/10.1029/2019GL082526 (2019).
6. Seroussi, H. *et al.* ISMIP6 Antarctica: a multi-model ensemble of the Antarctic ice sheet evolution over the 21st century. *The Cryosphere* **14**, 3033–3070, DOI: 10.5194/tc-14-3033-2020 (2020).
7. Seroussi, H. *et al.* Evolution of the Antarctic Ice Sheet Over the Next Three Centuries From an ISMIP6 Model Ensemble. *Earth's Futur.* **12**, e2024EF004561, DOI: https://doi.org/10.1029/2024EF004561 (2024).
8. Sun, S. *et al.* Antarctic ice sheet response to sudden and sustained ice-shelf collapse (ABUMIP). *J. Glaciol.* **66**, 891–904, DOI: 10.1017/jog.2020.67 (2020).
9. Morlighem, M. *et al.* Deep glacial troughs and stabilizing ridges unveiled beneath the margins of the Antarctic ice sheet. *Nat. Geosci.* **13**, 132–137, DOI: 10.1038/s41561-019-0510-8 (2020).
10. Mouginot, J., Rignot, E. & Scheuchl, B. Continent-Wide, Interferometric SAR Phase, Mapping of Antarctic Ice Velocity. *Geophys. Res. Lett.* **46**, 9710–9718, DOI: https://doi.org/10.1029/2019GL083826 (2019).
11. Rignot, E. *et al.* Four decades of Antarctic Ice Sheet mass balance from 1979–2017. *Proc. Natl. Acad. Sci.* **116**, 1095–1103, DOI: 10.1073/pnas.1812883116 (2019).
